# Fecal *Coprococcus*, hidden behind abdominal symptoms in patients with small intestinal bacterial overgrowth

**DOI:** 10.1186/s12967-024-05316-2

**Published:** 2024-05-25

**Authors:** Huaizhu Guo, Yuzhu Chen, Wenxin Dong, Siqi Lu, Yanlin Du, Liping Duan

**Affiliations:** 1 Department of Gastroenterology, Peking University Third Hospital, Beijing, China; 2 Department of Pediatrics, Peking University Third Hospital, Beijing, China; 3 International Institute of Population Health, Peking University Health Science Center, Beijing, China

**Keywords:** Small intestinal bacterial overgrowth, Hydrogen and methane breath test, Gut microbiome, Network analysis, Saccharolytic bacteria

## Abstract

**Background:**

Small intestinal bacterial overgrowth (SIBO) is the presence of an abnormally excessive amount of bacterial colonization in the small bowel. Hydrogen and methane breath test has been widely applied as a non-invasive method for SIBO. However, the positive breath test representative of bacterial overgrowth could also be detected in asymptomatic individuals.

**Methods:**

To explore the relationship between clinical symptoms and gut dysbiosis, and find potential fecal biomarkers for SIBO, we compared the microbial profiles between SIBO subjects with positive breath test but without abdominal symptoms (PBT) and healthy controls (HC) using 16S rRNA amplicon sequencing.

**Results:**

Fecal samples were collected from 63 SIBO who complained of diarrhea, distension, constipation, or abdominal pain, 36 PBT, and 55 HC. For alpha diversity, the Shannon index of community diversity on the genus level showed a tendency for a slight increase in SIBO, while the Shannon index on the predicted function was significantly decreased in SIBO. On the genus level, significantly decreased *Bacteroides*, increased *Coprococcus_2*, and unique *Butyrivibrio* were observed in SIBO. There was a significant positive correlation between saccharolytic *Coprococcus_2* and the severity of abdominal symptoms. Differently, the unique *Veillonella* in the PBT group was related to amino acid fermentation. Interestingly, the co-occurrence network density of PBT was larger than SIBO, which indicates a complicated interaction of genera. *Coprococcus_2* showed one of the largest betweenness centrality in both SIBO and PBT microbiota networks. Pathway analysis based on the Kyoto Encyclopedia of Genes and Genome (KEGG) database reflected that one carbon pool by folate and multiple amino acid metabolism were significantly down in SIBO.

**Conclusions:**

This study provides valuable insights into the fecal microbiota composition and predicted metabolic functional changes in patients with SIBO. *Butyrivibrio* and *Coprococcus_2*, both renowned for their role in carbohydrate fermenters and gas production, contributed significantly to the symptoms of the patients. *Coprococcus*’s abundance hints at its use as a SIBO marker. Asymptomatic PBT individuals show a different microbiome, rich in *Veillonella*. PBT’s complex microbial interactions might stabilize the intestinal ecosystem, but further study is needed due to the core microbiota similarities with SIBO. Predicted folate and amino acid metabolism reductions in SIBO merit additional validation.

**Supplementary Information:**

The online version contains supplementary material available at 10.1186/s12967-024-05316-2.

## Introduction

Small intestinal bacterial overgrowth (SIBO) is a sparsely recognized clinical syndrome as the presence of an abnormally excessive amount of bacterial colonization in the small bowel with abdominal complaints [1, 2]. SIBO is closely associated with many gastrointestinal (GI) diseases, such as irritable bowel syndrome (IBS) [3], inflammatory bowel disease (IBD) [4], pancreatitis [5], nonalcoholic liver disease [6], colorectal cancer and abdominal surgery [7]. It is believed that symptoms linked to SIBO consist of bloating, diarrhea, and abdominal pain/discomfort. Besides the main complaints, steatorrhea, vitamin B_12_ deficiency, and malnutrition can be seen in more severe cases [1]. Primary or secondary motility abnormalities destroy the ability of the small intestine to prevent colon bacterial translocation [8]. Meanwhile, ileocaecal valve dysfunction leads to colonic bacterial regurgitation [9]. Long-term medication of proton pump inhibitors (PPIs) is associated with an increased risk of SIBO. The intragastric defense barrier damage by acid suppression therapy makes it easier for upstream opportunistic pathogens to enter the small intestine [10–12]. Congenital or postoperative intestinal anatomical malformations increase local food residues and bacterial accumulation, like intestinal diverticulum, Roux-en-Y anastomosis, or small bowel resection [13, 14]. Multiple pathophysiological mechanisms contribute to abdominal discomforts including carbohydrate fermentation and improper metabolites, GI chronic inflammation, mucosal immune deficiency, increased gut permeability, food intolerance, and antigenemia [15, 16].

The gold standard for diagnosis of SIBO is a quantitative culture of small intestine aspirates. American Gastroenterological Association (AGA) recommended a new threshold at > 10^3^ colony-forming units per milliliter (CFU/mL) on fresh aspirate culture instead of > 10^5^ CFU/mL based on a large-scale study [17], derived from subjects with altered intestinal anatomy because the bacterial levels in normal subjects rarely exceed 10^2^ CFU/mL [1]. Based on the above diagnostic standard, SIBO subjects had a higher relative abundance of Proteobacteria and lower Firmicutes than non-SIBO subjects from the REIMAGINE study [18, 19]. Barlow et al. found that absolute loads of taxa in duodenal aspirates including *Klebsiella*, *Escherichia*, *Enterococcus*, and *Clostridium* enriched in individuals with SIBO were associated with more severe upper GI symptoms, but they lacked healthy controls [20]. Another cohort study found that duodenal aspirate microbiota was altered in symptomatic patients, while the absolute counting of anaerobes in the small intestine fluid wasn’t parallel with the severity of symptoms [21]. A significant proportion of patients deny effective treatment due to similar clinical phenotypes compared with many other functional gastrointestinal diseases (FGIDs). Few studies provide independent clinical and microbiome profiles of SIBO patients [22].

Indeed, the microbiological analysis from the small bowel fluid has limited clinical application considering that sampling is invasive and prone to contamination. An alternative method is the measurement of exhaled hydrogen and methane gas during the breath test (BT), which is considered a non-invasive, safe, useful, and cost-efficient method for SIBO. The North American Consensus recommended that a rise in hydrogen of ≥ 20 parts per million (ppm) or methane levels ≥ 10 ppm by 90 min during a glucose or lactulose breath test was considered positive [23]. However, the positive lactulose or glucose breath test representative of bacterial overgrowth could also be detected in asymptomatic subjects with the prevalence varying from 3 to 30% [24–28]. At present, we are still not clear about the possible mechanisms that lead to GI complaints and carbohydrate malabsorption in part of the positive BT population. Thus, the characterization of gut microbiota in SIBO patients with evident symptoms may provide a microbiological explanation for their clinical manifestations. This study aimed to illustrate the clinical characteristics and gut microbiota features of SIBO patients compared with asymptomatic positive breath test (PBT) subjects and health control (HC), as well as the correlation between clinical symptoms and microbiota alterations in SIBO. Through the comparison to asymptomatic PBT individuals, this study aimed to potentially explain the occurrence of symptoms in SIBO patients from the perspective of bacterial alterations. We expected to identify potential fecal biomarkers related to abdominal discomforts for SIBO patients.

## Patients and methods

### Patient recruitment

The study was performed from April 2019 to May 2021. SIBO patients who reported non-specific abdominal symptoms and fulfilled the diagnostic criteria of lactulose hydrogen and methane breath test were recruited from the Department of Gastroenterology, Peking University Third Hospital. The inclusion criteria of SIBO patients were as follows: (a) aged ≥ 18 and ≤ 65 years old; (b) GI discomfort, mainly abdominal pain, distension, constipation, or diarrhea for over 6 months; (c) positive lactulose hydrogen methane breath test (LBT); (d) voluntarily joined the study and completed the case report form, hydrogen methane breath test, and stool collection. Patients were excluded if they fulfilled one or more of the following exclusion criteria: (a) GI organic diseases detected by endoscopy or digestive tract surgery history; (b) with severe heart, liver, lung, kidney, blood, endocrine and nervous system diseases or severe respiratory tract, digestive tract, urinary tract infections or mental disorders; (c) taking antibiotics and acid suppression drugs for more than 3 days during the past month or probiotics, laxatives, antidiarrheal or prokinetic agents within 2 weeks; (d) pregnant or lactating women.

Asymptomatic subjects were recruited through advertising in the same clinical center. The exclusion criteria were: (a) less than 18 or more than 65 years old; (b) history of taking antibiotics, anti-diarrheal medications, laxatives, or seeking medical advice due to severe abdominal symptoms in the past 6 months; (c) a confirmed diagnosis of acute gastroenteritis in the past year; (d) taking probiotics or prebiotics in the past 2 weeks. According to their LBT results, they were divided into positive breath test (PBT) group and negative health control (HC).

### Clinical evaluation

Demographic data including age, gender, height, weight, and body mass index (BMI) were recorded for each participant. Daily bowel habit and frequency were recorded based on the Bristol stool form (BSF) scale [29]. GI symptom severity was evaluated by gastrointestinal symptom rating scale (GSRS) [30]. The symptom score was the sum of abdominal pain, distension, constipation, diarrhea scores. Self-rating anxiety scale (SAS) and self-rating depression scale (SDS) were used to evaluate the mental health conditions [31, 32]. 7-day food frequency questionnaire (FFQ) was used to estimate their dietary pattern [33]. Written informed consent was obtained from each participant prior to sample collection.

### Lactulose hydrogen and methane breath test (LBT)

All subjects were asked to refrain from antibiotic use and discontinue probiotics, laxatives, antidiarrheal, and prokinetic agents for 2 weeks. To minimize basal hydrogen excretion, dietary restriction and avoidance of smoking for at least 24 h before the test and during the test were recommended. Further, patients were asked to avoid coarse grains, milk, juice, and alcohol in the evening before the test. Fasting for 8 to 12 h before the procedure was required. Before the examination, subjects used 20 mL of antiseptic mouthwash (0.05% chlorhexidine) to eliminate fermentation by oral bacteria. End-expiratory breath samples were collected just before the ingestion of 10 g (15 mL) of lactulose in a 250 mL water solution. Gas samples were collected every 15 min until 90 min using the methane-hydrogen breathing analyzer DA6000 (Sunvou Medical Electronics Co., Ltd., Wuxi, China) which could test hydrogen, methane, hydrogen sulfide, oxygen, and carbon oxide immediately. The North American Consensus recommended that a rise in hydrogen of ≥ 20 ppm or methane levels ≥ 10 ppm by 90 min during a glucose or lactulose breath test was considered positive. A negative LBT was defined by none of the above criteria.

### Clinical feature statistical analysis

The analysis was conducted through SPSS V.26.0. The quantitative and qualitative variables were reported as mean ± standard error (SE), median ± interquartile range (IQR), and number (frequency). One-way analysis of variance (ANOVA) examined differences among groups for variables with normally distributed continuous variables, followed by a Fisher’s least significant difference (LSD) multiple comparisons post-test. Kruskal–Wallis non-parametric test examined differences among groups for discontinuous variables. χ^2^ test examined differences for categorical variables. Linear correlations were conducted through Pearson’s correlation analysis. Correlations for non-parametric variables were conducted through Spearman’s correlation analysis. A *p* < 0.05 was considered statistical significance for the above tests.

### Stool sampling

All included participants were required to stop using antibiotics, probiotics, prebiotics, and other microbiota-related supplements at least 2 weeks before stool sampling. Stool specimens were stored by stool nucleic acid collection tubes (Norgen Biotek Corp., Toronto, Ontario, Canada), then transported to the laboratory using dry ice and were frozen at − 80 °C.

### DNA extraction, PCR amplification, and sequencing

The sterile water blank samples were made as the control articles. DNA was extracted using E.Z.N.A.® soil DNA Kit (Omega Bio-tek, Norcross, GA, U.S.) according to the manufacturer’s instructions. The extracted genomic DNA of each sample was detected using 1% agarose gel electrophoresis. Two samples from the HC group were excluded because of low DNA loads. The hypervariable region V3–V4 of the bacterial 16S rRNA gene was amplified with primer pairs 338F (5′-ACTCCTACGGGAGGCAGCAG-3′) and 806R (5′-GGACTACHVGGGTWTTAAT-3′) by an ABI GeneAmp® 9700 PCR thermocycler (ABI, CA, U.S.). The PCR amplification of 16S rRNA gene was performed as follows: initial denaturation at 95 °C for 3 min, followed by 27 cycles of denaturing at 95 °C for 30 s, annealing at 55 °C for 30 s, extension at 72 °C for 45 s, single extension at 72 °C for 10 min, and ending at 4 °C. Purified amplicons were pooled in equimolar and paired-end sequences on an Illumina MiSeq PE300 platform (Illumina, San Degio, U.S.) according to the standard protocols by Majorbio Bio-Pharm Technology Co. Ltd. (Shanghai, China). A total of 6,789,850 16S rRNA sequences were obtained from the V3–V4 regions, with an average length of 416 bp per read.

### Gut microbiome sequencing data processing

The raw 16S rRNA gene sequencing reads were demultiplexed, quality-filtered by Fastq (version 0.20.0), and merged by Flash (version 1.2.7) with the following criteria: (1) the 300 bp reads were truncated at any site receiving an average quality score of < 20 over a 50 bp sliding window, the truncated reads shorter than 50 bp were discarded and reads containing ambiguous characters were also discarded; (2) only overlapping sequences longer than 10 bp were assembled according to their overlapped sequence; the maximum mismatch ratio of overlap region is 0.2; and reads that could not be assembled were discarded; (3) samples were distinguished according to the barcode and primers, and the sequence direction was adjusted, with exact barcode matching, and 2 nucleotide mismatch in primer matching.

The microbiome data profiling was performed by Parallel-Meta Suite (PMS, version 3.7) [34]. For details, all preprocessed 16S rRNA gene sequences were aligned with a 97% similarity level to the SILVA database (version 132) [35] for taxonomical annotation. Considering that the uneven sequencing depth (number of sequences) among samples may introduce bias (Table S1), we did sequence rarefaction for sequencing depth normalization after the taxonomic profiling. Using the PM-pipeline command in the PMS toolkit, set parameters as “-s 25,000” to enable this function and set the sequence depth to 25,000. The relative abundance of community members on each taxonomy level was also calculated by PMS. It first determines the copy number of each marker gene using the Integrated Microbial Genomes (IMG) database [36], and then corrects the relative abundance based on marker gene copy number normalization. This feature was enabled by default in PMS, and can be manually activated by inputting the parameter “-r T”. After taxonomic annotation of the operational taxonomy units (OTU), several sequencing quality control analyses were performed to verify the appropriate sequencing numbers and depth (Figure S1). The functional profiles were predicted with PICRUSt2 (Phylogenetic Investigation of Communities by Reconstruction of Unobserved States) and annotated with the Kyoto Encyclopedia of Genes and Genome (KEGG) Orthology (KO) [37].

### Diversity and abundance analysis of gut microbiome

For alpha diversity, Shannon indexes of each sample were calculated using “Vegan” package in R and tested by the Kruskal–Wallis rank sum test among three groups. A pairwise comparison was measured by the Wilcoxon rank sum test and false discovery rate (FDR) correction. For beta diversity, we utilized principal coordinates analysis (PCoA) based on the meta storms distance algorithm [38] and partial least squares discriminant analysis (PLS-DA). The computation and visualization were implemented using “vegan”, “mixOmics”, and “ggplot2” packages in R (version 4.2.1).

Then we screened genera with an average relative abundance exceeding 0.1% within each group considering them as commonly present in the respective group. (e.g., commonly present in the HC group). Subsequently, we visualized the unique genera within each group and the shared genera between groups using “Venn Diagram” package in R. This setup aims to prevent the identification of unique genera that exhibit significant individual differences and lack universality across the entire group. The differentially abundant genera and KEGG BRITE Level 3 pathways were examined by permutational multivariate analysis of variance (PERMANOVA), and their distribution was displayed using violin plots.

### Correlation and co-occurrence network analysis

The correlation of taxonomy and function that differed significantly among groups and clinical characteristics was quantitatively assessed using Spearman correlation analysis (“Hmisc” package in R). Then heatmaps were employed to illustrate correlations, where red indicates a positive correlation, blue indicates a negative correlation and darker colors represent larger |r| values, *p < 0.05, **p < 0.01, ***p < 0.001.

The co-occurrence networks were constructed based on Spearman correlation analysis between genera with a significance threshold of p < 0.05 and |r| ≥ 0.5. We used Cytoscape [39] (version 3.9.1) to visualize the network and calculate its topological properties.

Degree measures the total number of edges that connect to a node. Therefore, a node with a high degree will have a significant role in the network.

Network density represents the proportion of actual present connections in all possible relationships. The value is 0 if no connections are built in the network. The value closer to 1, the network is denser and the node is more cohesive in the network. Mathematically, $d(i)$ is the degree of the node i in the network. The equation of the density of the network nodes:

$$\rho =\frac{{\sum }_{i=1}^{N}d(i)}{N(N-1)}.$$

Betweenness centrality for each node is the number of these shortest paths that pass through this node, which represents the capacity to connect two or more non-adjacent nodes. The node with a higher betweenness centrality will have more control over the network. Let ${\sigma }_{st}$ be the total number of shortest paths from node *s* to node *t* and ${\sigma }_{st(i)}$ is the number of those paths that pass through *i*. The equation of the betweenness centrality of the node *i* is:

$$\text{B}\left(i\right)=\sum_{s\ne t\ne i}^{N}\frac{{\sigma }_{st(i)}}{{\sigma }_{st}}.$$

The Euclidean distance (De) of the betweenness centrality between networks P and Q in n-dimension space is calculated as:

$$De\left(P, Q\right)=\sqrt{\sum_{i=1}^{n}{({P}_{i}-{Q}_{i})}^{2}}.$$

## Results

### The clinical manifestation of SIBO patients

**Fig. 1:**
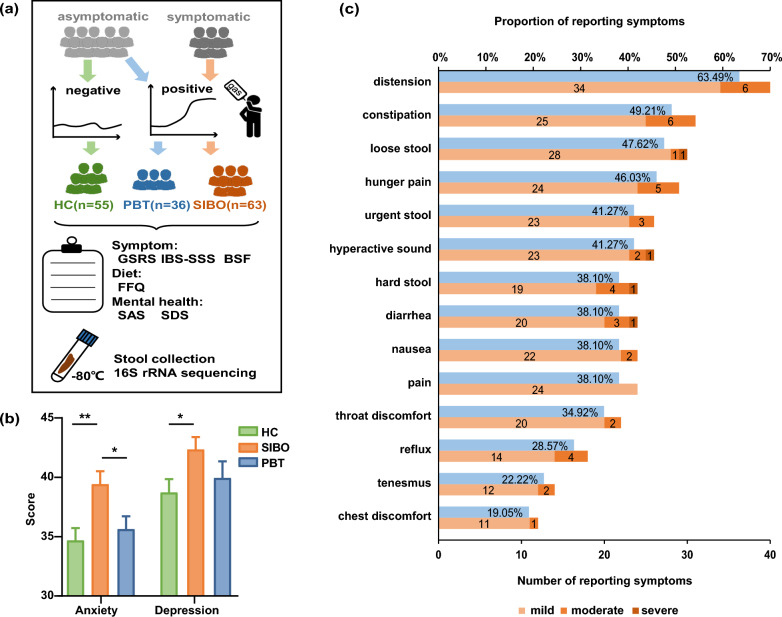
The flow diagram and clinical manifestations. **a** Flow diagram of the participants in this study. **b** Comparison of anxiety and depression scores. **c** The severity and proportion of gastrointestinal symptom rating scale (GSRS) distribution in SIBO patients. *HC* health control, *PBT* positive breath test, *SIBO* small intestinal bacterial overgrowth, *IBS-SSS* irritable bowel syndrome symptom severity scale, *BSF* Bristol stool form. *p < 0.05; **p < 0.01

In total, 154 subjects were enrolled, including 63 SIBO patients, 36 PBT, and 55 HC (Fig. 1a). There were no statistically significant differences among three groups in terms of gender, age, body mass index (BMI), carbohydrate, protein, fat consumption, and energy proportion (Table 1). No significant diet nutrient differences were found among three groups (Table S2). Patients with SIBO had significantly higher anxiety scores (39.42 ± 8.70) than PBT (36.53 ± 6.49, *p* < 0.05) and HC (34.68 ± 7.68, *p* < 0.01), respectively. The depression scores of SIBO (42.84 ± 8.31) were higher than that of HC (38.72 ± 8.41, *p* < 0.05) (Table 1 and Fig. 1b).

**Table 1:** Comparison of clinical manifestation among SIBO, PBT and HC groups

	HC	PBT	SIBO	P value
Number	55	36	63	
Sex (female, n/%)	32/58.18%	29/80.55%	45/71.42%	0.065
Age (year)	21.49 ± 2.08	22.08 ± 2.67	21.44 ± 2.72	0.431
BMI (kg/m^2^)	21.30 ± 2.79	21.44 ± 2.91	20.96 ± 2.38	0.635
Carbohydrate (g/d)	307.79 ± 43.13	223.57 ± 21.56	301.30 ± 58.21	0.494
Protein (g/d)	66.98 ± 6.01	59.35 ± 6.35	70.64 ± 7.22	0.847
Fat (g/d)	72.01 ± 7.36	67.89 ± 4.54	67.69 ± 3.80	0.541
Energy proportion of carbohydrate (%)	58.70 ± 1.83	53.77 ± 2.09	55.46 ± 1.48	0.160
Energy proportion of protein (%)	13.87 ± 0.51	14.09 ± 0.64	14.62 ± 0.54	0.523
Energy proportion of fat (%)	34.32 ± 2.44	36.90 ± 2.18	33.55 ± 1.32	0.590
Anxiety	34.68 ± 7.68	35.63 ± 6.49	39.42 ± 8.70^a,b^	< 0.01
Depression	38.72 ± 8.41	39.86 ± 8.91	42.84 ± 8.31^a^	< 0.05

^a^A significant difference compared with HC

^b^A significant difference compared with PBT

According to GSRS scores, the dominant symptoms in SIBO patients were distension (63.49%), constipation (49.21%), loose stool (47.62%), hunger pain (46.03%), urgent stool (41.27%), hyperactive sound (41.27%) and abdominal pain (38.10%) (Fig. 1c).

### Overall fecal microbiota composition and diversity

**Fig. 2:**
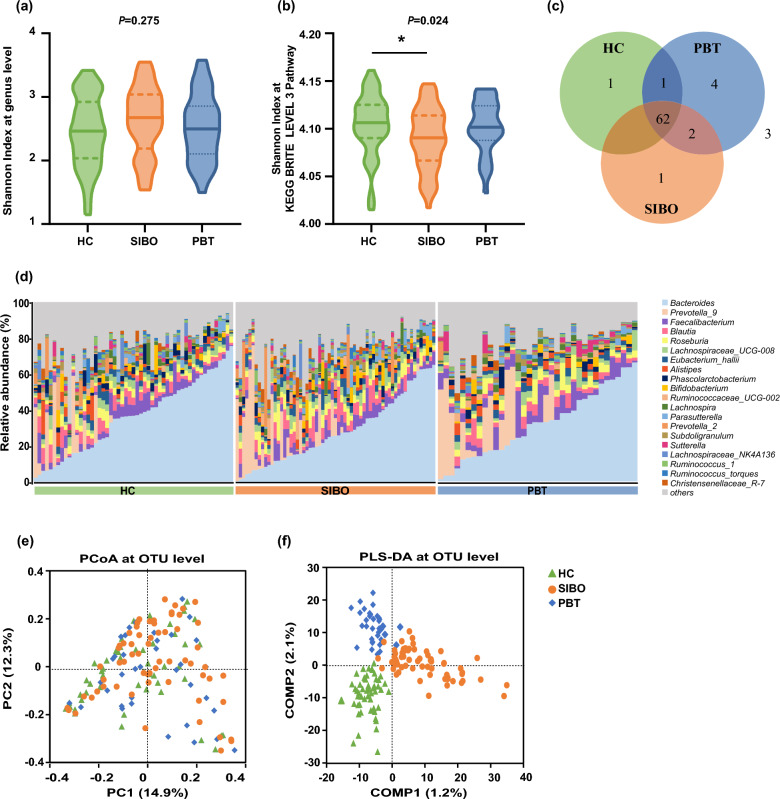
Overall microbiota composition and diversity. **a** Shannon index on the genus level of the alpha diversity. **b** Shannon index on the KEGG Level3 pathway. **c** Venn analysis. **d** The relative abundance distribution at the genus level. **e** PCoA at the OTU level of the beta-diversity analysis. **f** PLS-DA analysis at the OTU level. *KEGG* Kyoto encyclopedia of genes and genome, *PCoA* principal co-ordinates analysis, *OTU* operational taxonomic unit, *PLS-DA* partial least squares discriminant analysis. *p < 0.05

Even though no significant difference was found in the Shannon index of community diversity on the genus level, it showed a tendency for a slight increase in SIBO (*p* = 0.275, Fig. 2a). Notably, the Shannon index of SIBO significantly decreased compared with HC on the KEGG BRITE level3 pathway (*p* = 0.024, Fig. 2b). *Butyrivibrio* only occurred in SIBO, *Veillonella*, *Barnesiella*, *Escherichia–Shigella*, and *Tyzzerella_3* in PBT, and *Holdemanella* in HC, respectively. *Alloprevotella* and *Ruminiclostridium_6* were detected in both SIBO and PBT groups (Fig. 2c and Table 2). Each group was dominated by *Bacteroides,* followed by *Prevotella_9, Faecalibacterium, Blautia*, and *Roseburia* on the genus level (Fig. 2d). PCoA did not reveal any distinct clustering (Fig. 2e), while PLS-DA indicated a compositional separation of microbiota among three groups (Fig. 2f).

**Table 2:** The unique bacterial genera in Venn analysis and their metabolic characteristics derived from literatures

	Genus	Phylum	Family	Product	Gas
PBT	*Veillonella*	Firmicutes	Veillonellaceae	Polyamines, acetate, propionate [41, 42]	Yes [68]
	*Barnesiella*	Bacteroidota	Barnesiellaceae	Acetate succinate [69]	NR
	*Escherichia–Shigella*	Proteobacteria	Enterobacteriaceae	NR	NR
	*Tyzzerella_3*	Firmicutes	Lachnospiraceae	NR	NR
PBT&SIBO	*Alloprevotella*	Bacteroidota	Prevotellaceae	Acetate succinate [77]	Yes [77]
	*Ruminiclostridium_6*	Firmicutes	Oscillospiraceae	Acetate, propionate, butyrate [78]	Yes [78]
SIBO	*Butyrivibrio*	Firmicutes	Lachnospiraceae	Butyrate [51, 52]	Yes [53]
HC	*Holdemanella*	Firmicutes	Erysipelotrichaceae	Acetate, propionate, butyrate, lactic acid [79]	NR

*PBT* positive breath test, *SIBO* small intestinal bacterial overgrowth, *HC* health control, *NR* no related evidence

### Fecal microbiota taxonomic changes for screening potential biomarkers

**Fig. 3:**
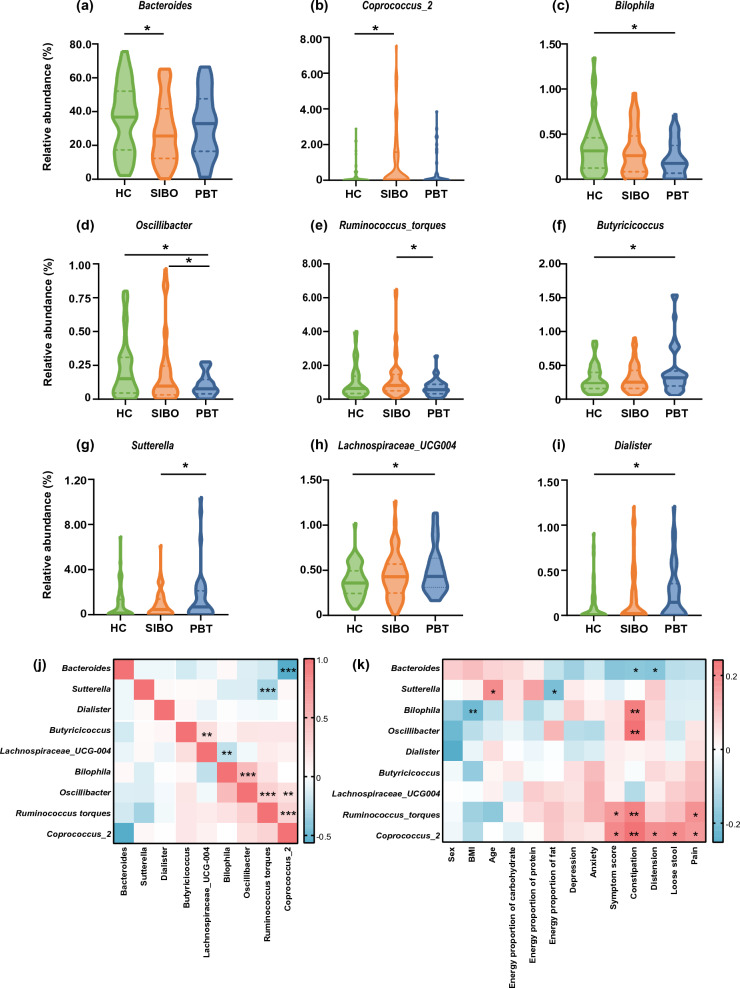
Different microbiota profiles and inner correlation. **a**–**i** The relative abundance of different genera. **j** The inner Spearman correlation heatmap on the genus level. **k** The Spearman correlation heatmap between microbiota and host factors. *p < 0.05; **p < 0.01; ***p < 0.001

A significantly lower abundance of *Bacteroides* and a higher abundance of *Coprococcus_2* were observed in SIBO compared with HC on the genus level (Fig. 3a, b). It's worth mentioning that a greater variety of microbiota differences were observed in the PBT group. The relative abundance of *Bilophila*, *Oscillibacter*, and *Ruminococcus_torques* was significantly decreased, and the *Butyricicoccus*, *Sutterella*, *Lachnospiraceae_UCG004*, and *Dialister* were enriched in PBT (Fig. 3c–i).

Furthermore, we assessed the Spearman’s correlations of these microbiota which suggested synergistic and competitive interactions (Fig. 3j). The relative abundance of *Bacteroides* was negatively associated with that of *Coprococcus_2* (*r* = − 0.563, *p* < 0.001). We also detected the negative correlation between *Ruminococcus_torques* and *Sutterella* (*r* = − 0.304, *p* < 0.001), as well as between *Bilophila* and *Lachnospiraceae_UCG004* (*r* = − 0.210, *p* < 0.01). The significantly positive correlations between each pair of the three decreasing genera of PBT suggested a similar trend. The relative abundance of *Coprococcus_2* was positively associated with that of *Oscillibacter* (*r* = 0.218, *p* < 0.01) and *Ruminococcus_torques* (*r* = 0.310, *p* < 0.001).

The correlation heatmap revealed significant positive correlations between the relative abundance of *Coprococcus_2* and the severity of all symptoms (Fig. 3k). *Bacteroides* was negatively related to constipation and distension. No significant relation was found between the differential genera and the mental scores.

### Microbial co-occurrence network analysis

**Fig. 4:**
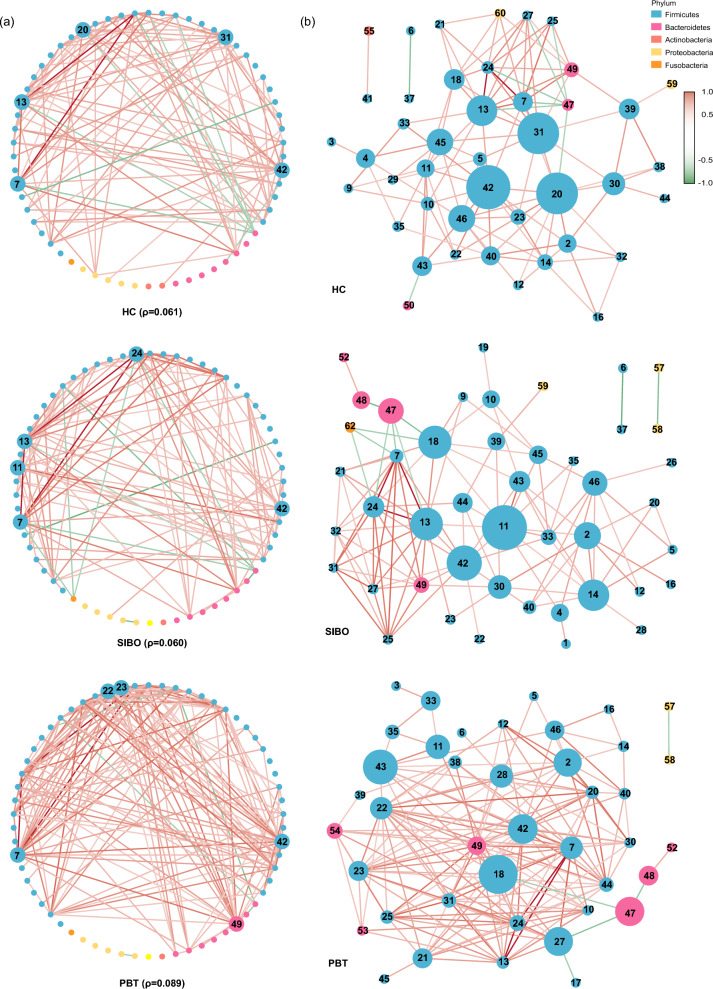
Co-occurrence network of relative abundance of sixty-two common genera in three groups. The absolute value of a relative coefficient greater than 0.5 was coded in the line color according to statistical significance (p < 0.05). where red indicates a positive correlation, blue indicates a negative correlation and darker colors represent larger |r| values. The color of each node refers to the phylum. The genus names corresponding to each node are shown in Table S3. **a** Degree calculated in the network. The largest five nodes indicated the highest degree in the network. **b** Betweenness centrality was calculated in the network. The size of each node was proportional to the betweenness centrality

The topological properties of each co-occurrence network were analyzed through degree and betweenness centrality. Figure 4a showed the largest network density in PBT (ρ = 0.089), which represented a more complex microbiota interaction compared to HC (ρ = 0.061) and SIBO (ρ = 0.060). The size of each node was proportional to the betweenness centrality in Fig. 4b. It was worth noting that *Coprococcus_2* (node 18) showed a high betweenness centrality, representing it was necessary for the connectivity in both SIBO and PBT networks. To verify the similarity of the network, we calculated the Euclidean distance of betweenness centralities. The distance between PBT and SIBO is 512.78, the distance between PBT and HC is 539.76, and the distance between HC and SIBO is 557.60, reflecting that the necessary microbiota of the whole network were more similar in PBT and SIBO.

### Diminishing metabolic functions associated with abdominal symptoms in SIBO

**Fig. 5:**
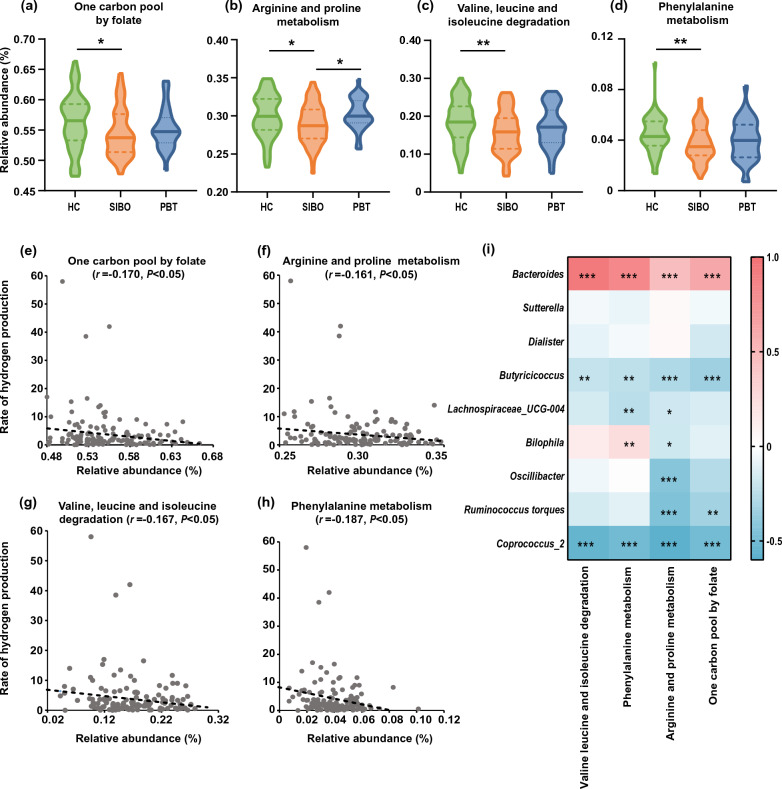
The predicted microbiota function alterations in SIBO patients. **a**–**d** The relative abundance on the KEGG pathways. **e**–**h** The scatter plot of the gas production and the function relative abundance. Axis Y presented the hydrogen elevation rate at 90 min compared to the baseline level. **i** The Spearman correlation heatmap of relative abundance between KEGG BRITE level 3 pathway and genera. *p < 0.05; **p < 0.01; ***p < 0.001

To explore the functional changes associated with differences in microbial composition, gut microbiome function was imputed using PICRUSt2 and pathway analysis based on the KEGG database (Fig. 5a–d). Interestingly, pathways associated with amino acid metabolism were down in SIBO, including arginine and proline metabolism, valine, leucine and isoleucine degradation, and phenylalanine metabolism, mostly essential amino acids involved. The pathway reflective of one carbon pool by folate was significantly dropped in SIBO. Furthermore, the significant negative correlations between the gas production rate at 90 min and the functions were stable (Fig. 5e–h). To determine the association between microbiota functional diversity and disease, the relative abundance of the above functions and host parameters were considered for the correlation analyses. Overall, the above predicted functional changes were negatively associated with symptom scale, constipation, abdominal distension, and pain (Figure S2). The predicted functional changes also had a significantly positive correlation with *Bacteroides* and a negative correlation with *Coprococcus_2*, supporting the synergism with the taxonomic relative abundance (Fig. 5i).

## Discussion

**Fig. 6:**
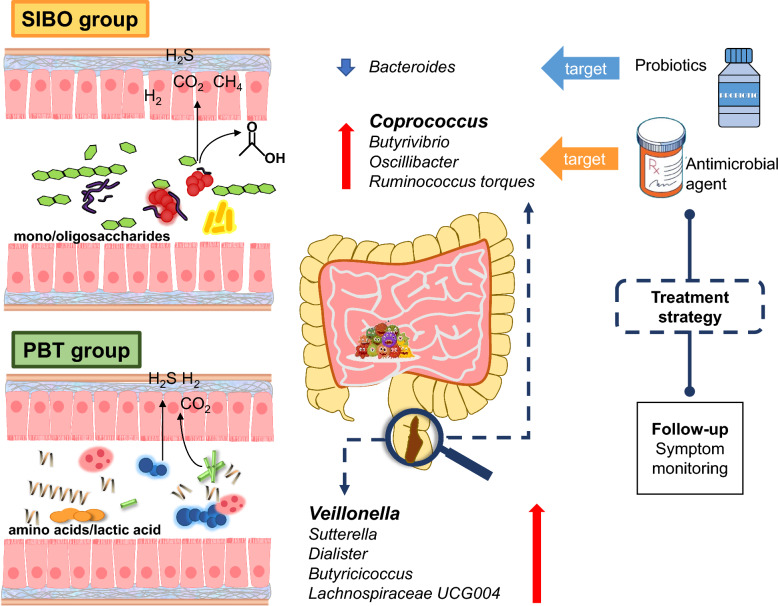
The probable explanation of the microbiota effect for SIBO and PBT group and potential treatment strategies. *PBT* positive breath test, *SIBO* small intestinal bacterial overgrowth

In this study, we present the microbial composition from the positive hydrogen and methane breath test population with and without abdominal discomfort and with an otherwise healthy gut for the first time. The upward trend of taxonomic diversity and decreasing functional diversity were consistent with the progression of SIBO. The increase in taxonomic diversity may be attributed to changes in the distribution of different microorganisms within the intestine. Some genera that were originally in low abundance have multiplied, and the dominant genera like *Bacteroides* have reduced (Figure S3a). On the other hand, the functions are highly similar and dominant functions extremely rise, leading to a reduction in overall functional diversity (Figure S3b). A significant decrease in *Bacteroides* and an increase in *Coprococcus_2* were observed, along with a unique occurrence of *Butyrivibrio* in SIBO, which has been reported to be associated with gas production through carbohydrate fermentation (Table 2) [40]. Pathway analysis based on the KEGG database reflected that one carbon pool by folate and amino acid metabolism were significantly down in SIBO. Both the composition and function alterations of the microbiota were correlated with GI symptoms in SIBO. On the other hand, a great variety of microbiota represented by *Veillonella* in PBT was associated with the fermentation of amino acids and peptides previously reported (Table 2) [41, 42]. Asymptomatic individuals with a solely positive breath test possessed a strongly connected network reflecting the more complicated interactions of the fecal microbiota (Fig. 6).

Mostly, SIBO patients complain of non-specific GI symptoms due to the presence of excessive colonization of aerobic or anaerobic bacteria in the small bowel [43]. The symptoms are closely related to the fermentation of non-absorbed carbohydrates like nausea, bloating, flatulence, distension, abdominal pain, diarrhea, and/or constipation [44]. A significant proportion of patients deny effective treatment as misdiagnosed as IBS due to the unclear symptom spectrum [2, 45, 46]. In our study, we found that the most frequently reported symptom was abdominal distension, followed by changes in defecation habits. Abdominal pain for the essential diagnosis of IBS in Rome IV consensus was not highlighted for SIBO patients. Gas-producing related symptoms such as bloating, gassiness, cramping, and distension were more obvious [47]. Primary or secondary motility abnormalities destroy the ability of the small intestine to prevent colon bacterial translocation [8], thus slow intestinal transit leads to excessive gas retention and constipation [48].

The small intestine represents the first region where food components and the intestinal bacteria encounter each other for primary carbohydrate metabolism. SIBO can be defined as the inappropriate fermentation of many kinds of carbohydrates and simultaneously multiple nutrient malabsorption detected by the culture of proximal intestinal aspirates or measurement of exhaled hydrogen and methane. Over the past few decades, the lack of knowledge about SIBO was confined to the collection, storage, and culture of small bowel fluids. Almost all samples were obtained near the duodenal or jejunum rather than the bacterial colonization by upper gastrointestinal endoscopy at the risk of contamination. Inevitably gas injection during the endoscopic operation may disturb the anaerobic culture of SIBO [49, 50]. The fecal microbiota composition alterations may help in the explanation of the metabolism features and progression of SIBO. The *Butyrivibrio* spp. from Lachnospriaceae detected only in SIBO patients could encode a more impressive repertoire of carbohydrate-active enzymes than most Firmicutes [40], capable of growing on a range of carbohydrates, from mono-or oligosaccharides to complex plant polysaccharides, such as pectins, mannans, starch, and hemicelluloses [51, 52]. The end products were butyrate and many kinds of gas including hydrogen (H_2_), carbon dioxide (CO_2_), and hydrogen sulfide (H_2_S). *Butyrivibrio* was significantly abundant in subjects that reported traveler’s diarrhea [53], while also significantly higher in the constipation-dominant IBS patients from mucosal samples [54].

Our findings supported a negative correlation between the relative abundance of *Bacteroides* and GI symptoms, concurred with a lower amount of *Bacteroides* in SIBO patients. Several species of *Bacteroides* which we considered beneficial bacteria could access their desired nutrients from long-chain polysaccharides and oligosaccharides that are not readily absorbed by the epithelial cells of the colon in healthy status based on the polysaccharide utilization loci (PULs) [55], producing useful short-chain fatty acids. Bamba et.al have found that the relative abundance of *Bacteroides* in duodenal aspirates of SIBO patients was significantly lower than that of non-SIBO patients, consistent with our findings [56]. Therefore, we inferred that the decline of *Bacteroides* in fecal samples in accordance with the duodenal aspirates reflected the overuse of carbohydrates or inner competition by proliferating bacteria in the small intestine.

Conversely, the genus *Coprococcus_2* was positively correlated with the symptom score and all symptoms concurred with a higher abundance in SIBO. *Coprococcus* spp. within the family, Lachnospiraceae of Firmicutes are deemed the core genera for the maintenance of microbial homeostasis and healthy status [57, 58], as they contribute to the production of the health-promoting metabolite butyrate. Nevertheless, it was reported that *Coprococcus_2* was associated with a higher risk of IBD, obesity, and polycystic ovary syndrome (PCOS) [59–61]. Several studies indicated the enrichment of *Coprococcus* in SSc, autism spectrum disorder, and radiation enteritis (RE) [62–64]. There are significant differences in the utilization of carbohydrates among *Coprococcus* subgroups. Multiple carbon source substrates could be utilized by *C.eutactus*, mainly contained in *Coprococcus_2* [65]. As a short-chain fatty acid-producing bacterium, *C. eutactus* mainly generates acetic acid [66]. However, we lack sufficient understanding of the impact of *Coprococcus* on SIBO. A randomized clinical trial of berberine and rifaximin effects for SIBO is underway in our clinical center [67]. The unpublished preliminary analysis verified the enrichment of *Coprococcus* in SIBO patients compared with healthy individuals again. We further found that a lower relative abundance of *Coprococcus* inhibited by berberine was observed in patients with negative hydrogen methane breath tests after medication compared with baseline (0.18 ± 0.13% vs. 1.09 ± 0.20%, *p* < 0.001). On the contrary, there was no significant change in the relative abundance of *Coprococcus* before and after medication in those who failed to respond to berberine (0.32 ± 0.17% vs. 0.27 ± 0.11%, *p* = 0.775). The baseline relative abundance of *Coprococcus* could also indicate drug response (Figure S4). The increased *Coprococcus* abundance may be one of the potential biomarkers of SIBO. Elimination of *Coprococcus* might be the key approach to eradicating bacterial colonization and helping patients achieve clinical improvement. Further studies should be performed to determine the disruptors in the small intestine.

Several taxonomic groups we identified in PBT reflected the diverse nutrient metabolic features as the highlight of this study. *Veillonella* from the family Veillonellaceae of Firmicutes existed in 28 of 36 PBT objects and were also commonly found in duodenal aspirate sequencing [20]. *Veillonella* is the predominant component in the small intestine of healthy subjects [68]. It is characterized in that glucose or any other carbohydrate is not fermented, but relies on organic acids, amino acids, and peptides as carbon sources which may explain the exhaled gas production like H_2_ and H_2_S (Table 2) [41, 42]. It was also reported that *Barnesiella*, another unique genus that occurred in most PBT individuals, could utilize amino acids and proteins as carbon sources [69]. Asaccharolytic *Dialister*, Enriched in PBT, had close phylogenetic distance and similar physiological characteristics with *Veillonella* and was overrepresented in cirrhosis duodenum [70, 71]. The great abundance of the above taxa also suggested higher transport and survival of oral microorganisms and promoted the growth of the upper gastrointestinal tract species in the distal bowel related to weight loss after gastric surgery in the previous study [72, 73]. However, in our study, they were observed in asymptomatic PBT individuals. It indicated that the existence of these taxa was impossibly responsible for inducing abdominal discomfort. A more stable network we observed in the PBT population provided a possible protective effect. The strong positive correlations between each pair of genera reveal that they grow and proliferate synchronously. In contrast, the negative correlations of the genus abundance indicate that they may compete with survival resources to inhibit each other. The more bacterial interactions in the intestinal microenvironment could help maintain gut homeostasis and make it less susceptible to being disturbed by external environmental factors. The genera with the high degree simply centralized in the Firmicutes in the SIBO group in contrast to *Bacteroides* from Bacteroidota participated in maintaining the stability of the network in PBT. The betweenness centrality distribution indicated that the essential “bridges” were distinct in each network. However, the similarity could be found in PBT and SIBO due to the smaller Euclidean distance which demonstrated potential pathogens like *Coprococcus_2* might be shared in two groups. In brief, we need to pay more attention to the healthy conditions of PBT people even though no abdominal discomfort has been reported so far.

PICRUSt2 analysis found that the metabolic function alterations matched with the microbiota abundance changes. Amino acid metabolism pathways, mostly essential amino acids involved, were downregulated in SIBO patients reflecting the result of competition of the nutrient metabolism in the small bowel. The identified bacteria in our study were previously reported as carbohydrate fermenters, consistent with observations in other studies. This finding may be attributed to the down-regulation of amino acid metabolism, potentially interfering with amino acid absorption. By contrasting our results with existing literature, we aim to elucidate the significance of our findings within the context of current research. In addition, biosynthesis and cycle of tetrahydrofolate were downregulated according to the decreased one carbon pool by folate in SIBO, which provided the potential explanation for megaloblastic anaemia in more severe patients [1]. The metabolic pathway functions were also negatively correlated with the symptom spectrum and hydrogen levels, which illustrated the harmful effects of SIBO on the microbiota metabolism function. The predicted metabolic function alterations in SIBO are worth further validation.

We also pay attention to the mental health of SIBO patients. Gut–brain–microbiota axis plays a core role in many FGIDs and provides a potential treatment target for mental disorders [74]. However, there was sparse knowledge about the mental status of SIBO patients. In our study, anxiety and depression scores in the SIBO group using self-reporting scales were worse compared with healthy individuals. Interestingly, the anxiety scores of symptomatic patients were also significantly higher compared with PBT, both with the positive breath test which represented similar intestinal microbial loads, which may indicate the participation of psychosocial abnormalities in SIBO. Neither anxiety nor depression scores were significantly related to the relative abundance of *Coprococcus_2*, which denied that the changes in gut microbiota might be caused by abnormal psychiatric status. We supposed that psychosocial abnormalities may be involved in abdominal complaints.

In this study, asymptomatic individuals with abnormal breath tests were recruited for the first time. The bacterial composition and functional characteristics compared by 16S rRNA sequencing revealed possible microorganisms for GI symptoms in hydrogen/methane-producing populations. The saccharolytic bacteria associated with the development of SIBO and functional abnormalities were found. There was a significant correlation between *Coprococcus_2* and the severity of symptoms, which may be one of the biomarkers of SIBO. We also adopted novel bioinformatics methods and innovatively applied statistical parameters to establish objective indicators of network analysis. This may provide a basis for targeted treatment of pathogenic bacteria of SIBO in the future.

However, it has some limitations which should not be neglected. First, we lack direct small intestine samples. Although the convenient and non-invasive fecal samples reflected the disturbed luminal contents influenced by the upstream bacterial overgrowth in our study, we need to take into account that the microbiota transmission from the small intestine to the colon could not behave consistently [75]. The small intestine fluid and mucosal biopsies might be more representative to reflect the local pathogenic microbiota and host interactions even though there is still debate about the sampling position and contamination [1, 75, 76]. Additionally, 16s rRNA sequencing could represent the existence and abundance of the microbes but lack the details of how these microbiota perform their functions. It is nowhere near enough to only provide the functional prediction analysis to reflect the actual role. Multi-omics analysis is necessary to help us understand the connection between the metabolic functions of microbes and the disease progression better. Finally, we failed to follow up on the symptoms of PBT individuals so the long-term impact of differential microbes in the small intestine is unknown. It deserves further concern about their future health status and whether they will develop GI symptoms with persistent intestinal dysbiosis.

## Conclusion

This study delivers significant understanding of the fecal microbiota composition and metabolic functional shifts anticipated in SIBO patients, elucidating the factors contributing to their abdominal discomfort. Notably, *Butyrivibrio* and *Coprococcus_2*, both known for gas production through carbohydrate fermenters, contributed significantly to the discomfort experienced by patients with SIBO. Furthermore, the enrichment of *Coprococcus* suggests its potential as a biomarker for SIBO. On the contrary, asymptomatic PBT subjects exhibited a distinct microbiome spectrum, represented by enriched *Veillonella*. The complicated network interactions of PBT might provide a stable intestinal environment, but it deserved further follow-up due to the similar core microbiota with SIBO. It is worth further validation that one carbon pool by folate and multiple amino acid metabolism were significantly down in SIBO based on the KEGG database.

### Supplementary Information

Supplementary Material 1: Figure S1. The taxonomy annotation analysis. (a) Rank-abundance curves. (b) Refraction curves. (c) Pan analysis. (d) Core analysis. HC: health control; PBT: positive breath test; SIBO: small intestinal bacterial overgrowth.

Supplementary Material 2: Figure S2. The Spearman correlation heatmap between functional changes and host factors. *p < 0.05; **p < 0.01; ***p < 0.001.

Supplementary Material 3: Figure S3. The numerical difference of the relative abundance between SIBO and HC. (a) The relative abundance of the taxonomic composition. (b) the relative abundance of the KEGG BRITE Level3 pathway. HC: health control; SIBO: small intestinal bacterial overgrowth.

Supplementary Material 4: Figure S4. The validation set of the relative abundance of *Coprococcus* in SIBO. (a) Compared with healthy individuals. (b) Before and after medication of berberine in responders and non-responders. *A significant difference before and after medication of berberine in responders; ^#^a significant difference at baseline in two groups. ***p < 0.001, ^##^*p* < 0.01.

Supplementary Material 5: Table S1. The sequencing depth among SIBO, PBT and HC groups.

Supplementary Material 6: Table S2. Comparison of diet nutrients among SIBO, PBT and HC groups.

Supplementary Material 7: Table S3. The node information of the co-occurrence network.

## Data Availability

The data that support the findings of this study are openly available in the National Center for Biotechnology Information Sequence Read Archive (SRA) repository at the reference number PRJNA907418.

## Abbreviations

AGA: American gastroenterological association
ANOVA: Analysis of variance
BMI: Body mass index
BSF: Bristol stool form
BT: Breath test
CFU: Colony-forming units
CO_2_: Carbon dioxide
FDR: False discovery rate
FFQ: Food frequency questionnaire
FGIDs: Functional gastrointestinal diseases
GI: Gastrointestinal
GSRS: Gastrointestinal symptom rating scale
H_2_S: Hydrogen sulfide
HC: Healthy controls
IBD: Inflammatory bowel disease
IBS: Irritable bowel syndrome
IQR: Interquartile range
KEGG: Kyoto Encyclopedia of Genes and Genome
KO: Kyoto Encyclopedia of Genes and Genome Orthology
LBT: Lactulose hydrogen methane breath test
OTU: Operational taxonomy units
PBT: Positive breath test
PCoA: Principal coordinates analysis
PCOS: Obesity and polycystic ovary syndrome
PERMANOVA: Permutational multivariate analysis of variance test
PLS-DA: Partial least squares discriminant analysis
PPIs: Proton pump inhibitors
ppm: Part per million
PULs: Polysaccharide utilization loci
SAS: Self-rating anxiety scale
SDS: Self-rating depression scale
SE: Standard error
SIBO: Small intestinal bacterial overgrowth
