# Supplementary figures and images for: Fecal Coprococcus, hidden behind abdominal symptoms in patients with small intestinal bacterial overgrowth
Source: J Transl Med. 2024 May 25;22:496. doi: 10.1186/s12967-024-05316-2 (PMC11128122; doi:10.1186/s12967-024-05316-2)

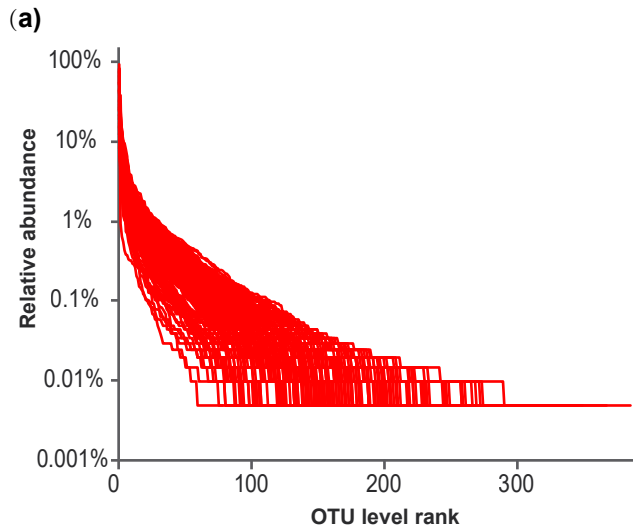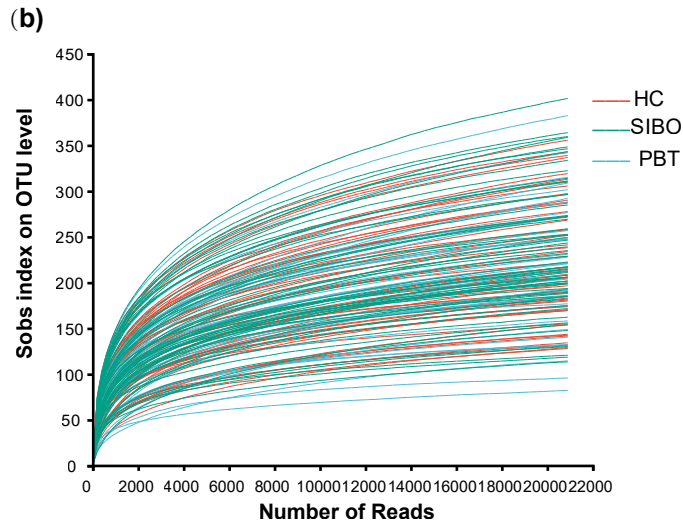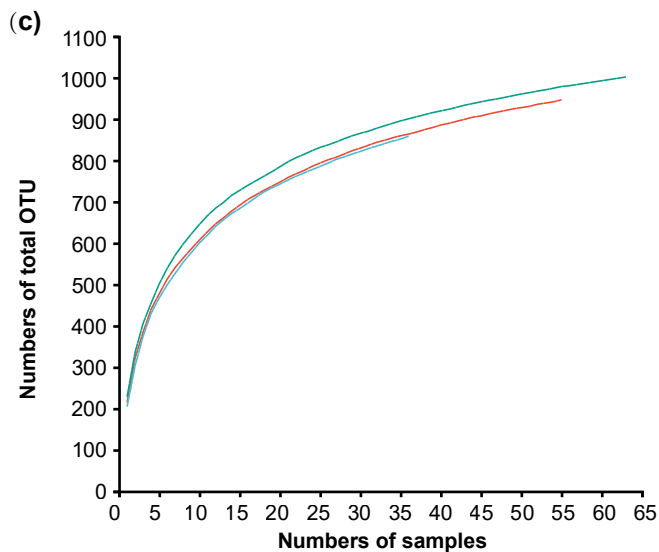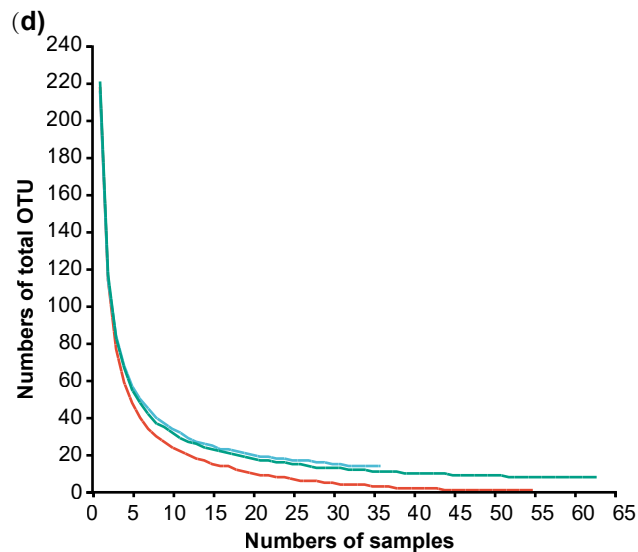

Supplement: Supplementary file 1 — Supplementary Material 1: Figure S1. The taxonomy annotation analysis. (a) Rank-abundance curves. (b) Refraction curves. (c) Pan analysis. (d) Core analysis. HC: health control; PBT: positive breath test; SIBO: small intestinal bacterial overgrowth. [file 12967_2024_5316_MOESM1_ESM.pdf]

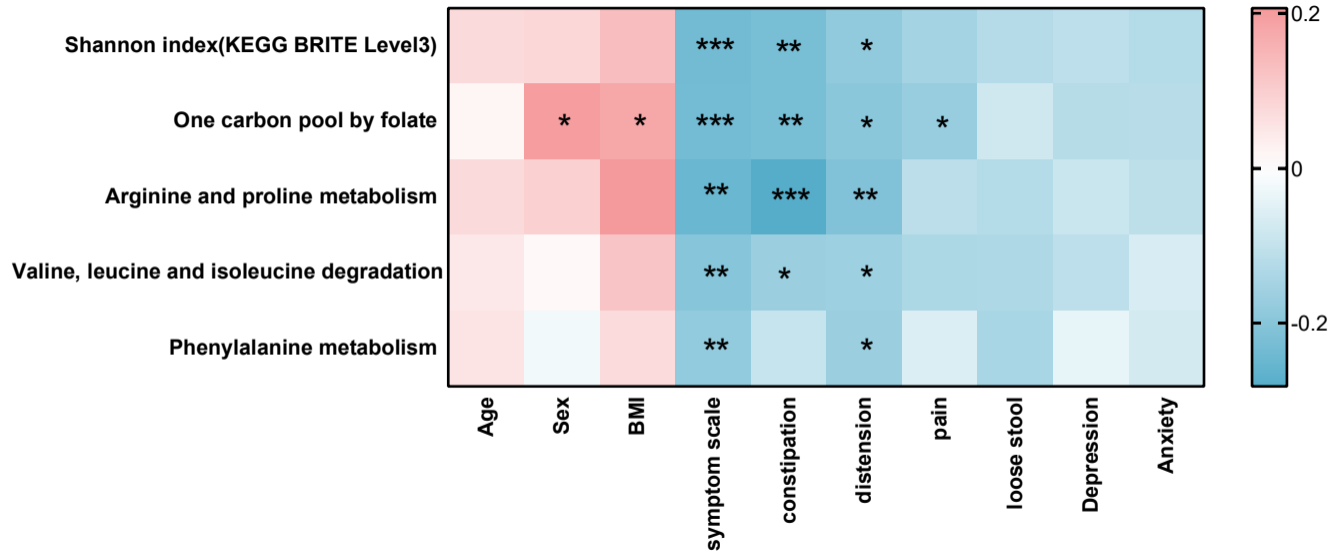

Supplement: Supplementary file 2 — Supplementary Material 2: Figure S2. The Spearman correlation heatmap between functional changes and host factors. *p < 0.05; **p < 0.01; ***p < 0.001. [file 12967_2024_5316_MOESM2_ESM.pdf]

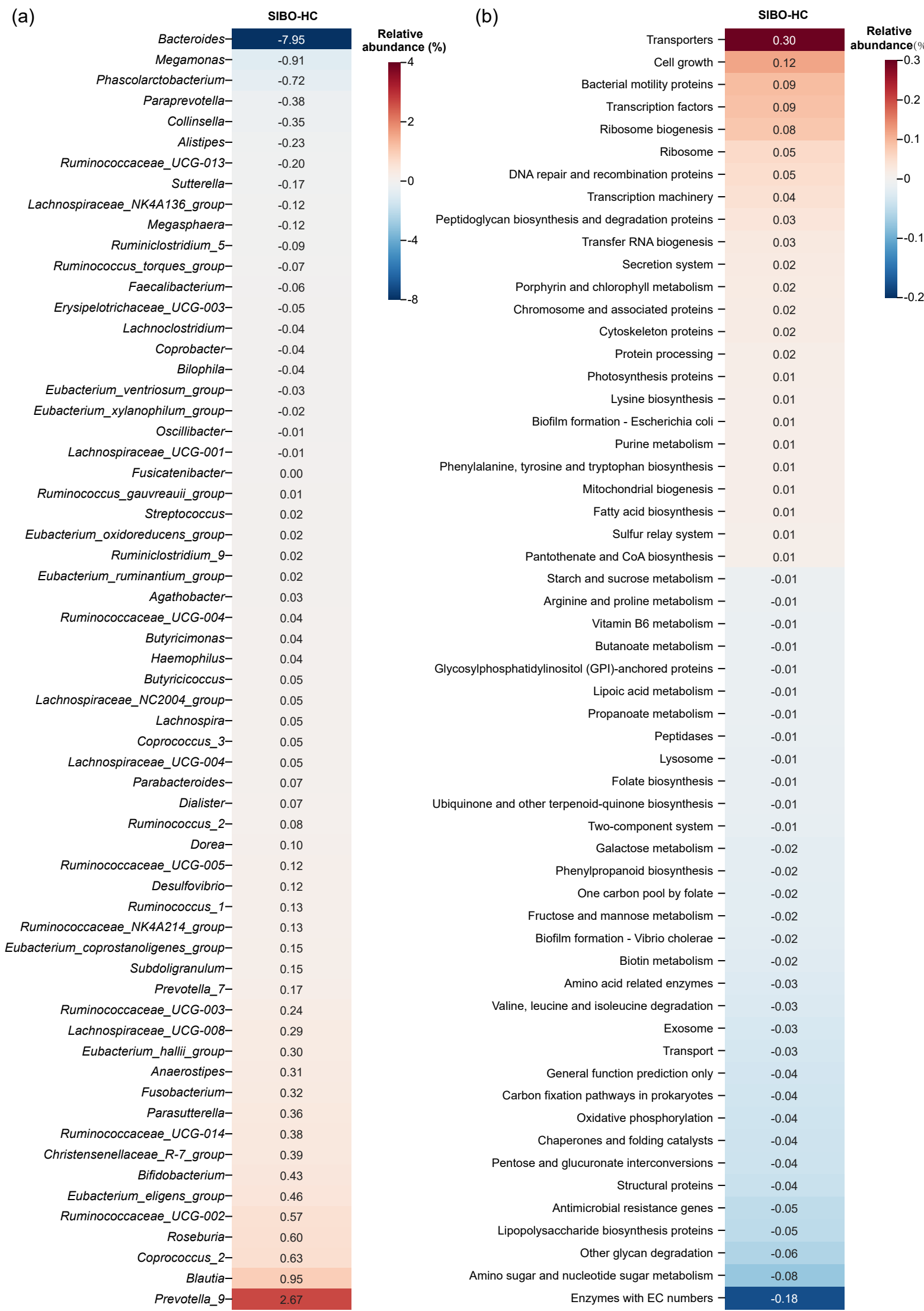

Supplement: Supplementary file 3 — Supplementary Material 3: Figure S3. The numerical difference of the relative abundance between SIBO and HC. (a) The relative abundance of the taxonomic composition. (b) the relative abundance of the KEGG BRITE Level3 pathway. HC: health control; SIBO: small intestinal bacterial overgrowth. [file 12967_2024_5316_MOESM3_ESM.pdf]

(a)

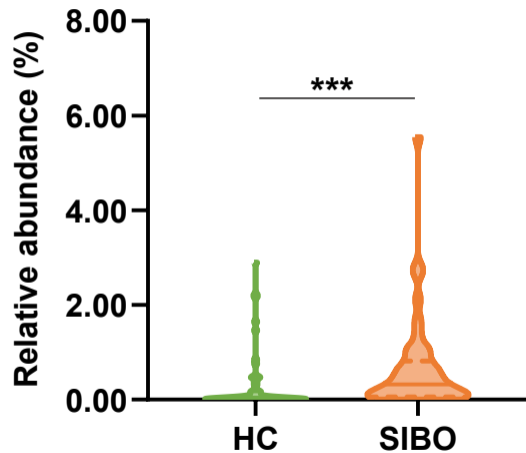

(b)

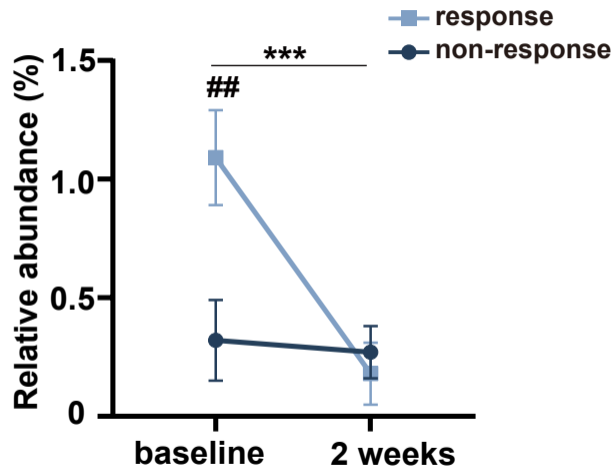

Supplement: Supplementary file 4 — Supplementary Material 4: Figure S4. The validation set of the relative abundance of Coprococcus in SIBO. (a) Compared with healthy individuals. (b) Before and after medication of berberine in responders and non-responders. *A significant difference before and after medication of berberine in responders; #a significant difference at baseline in two groups. ***p < 0.001, ##p < 0.01. [file 12967_2024_5316_MOESM4_ESM.pdf]
