# Supplementary material for: Fecal Coprococcus, hidden behind abdominal symptoms in patients with small intestinal bacterial overgrowth
Source: J Transl Med. 2024 May 25;22:496. doi: 10.1186/s12967-024-05316-2 (PMC11128122; doi:10.1186/s12967-024-05316-2)
Supplement: Supplementary file 6 — Supplementary Material 6: Table S2. Comparison of diet nutrients among SIBO, PBT and HC groups. [file 12967_2024_5316_MOESM6_ESM.docx]

Table S2 Comparison of diet nutrients among SIBO, PBT and HC groups

|  | HC | PBT | SIBO | *P* value |
| --- | --- | --- | --- | --- |
| Number | 55 | 36 | 63 |  |
| Dietary fiber (g/d) | 16.92±1.85 | 13.38±1.37 | 15.92±1.37 | 0.603 |
| Cholesterol (mg/d) | 291.59±27.73 | 306.16±28.16 | 439.96±120.70 | 0.445 |
| Vitamin A (ug/d) | 637.18±79.49 | 556.85±75.38 | 797.47±121.84 | 0.156 |
| Vitamin B12 (ug/d) | 4.65±1.48 | 12.82±4.77 | 7.84±2.09 | 0.204 |
| Folate (ug/d) | 18.88±2.63 | 26.35±4.84 | 21.99±3.82 | 0.610 |
| Vitamin C (mg/d) | 125.37±13.25 | 121.47±20.18 | 169.99±23.69 | 0.176 |
| Vitamin D (ug/d) | 0.77±0.08 | 1.00±0.11 | 1.00±0.37 | 0.731 |
| Vitamin E (mg/d) | 58.70±1.83 | 53.77±2.09 | 55.46±1.48 | 0.594 |
| Calcium (mg/d) | 417.27±35.88 | 374.10±33.93 | 432.93±35.60 | 0.436 |
| Iodine (ug/d) | 23.89±2.04 | 23.17±2.51 | 30.64±5.62 | 0.495 |
